# Intra-urban variability of long-term exposure to PM_2.5_ and NO_2_ in five cities in Colombia

**DOI:** 10.1007/s11356-023-31306-w

**Published:** 2023-12-12

**Authors:** Laura A. Rodriguez-Villamizar, Yurley Rojas, Sara Grisales, Sonia C. Mangones, Jhon J. Cáceres, Dayana M. Agudelo-Castañeda, Víctor Herrera, Diana Marín, Juan G. Piñeros Jiménez, Luis C. Belalcázar-Ceron, Oscar Alberto Rojas-Sánchez, Jonathan Ochoa Villegas, Leandro López, Oscar Mauricio Rojas, María C. Vicini, Wilson Salas, Ana Zuleima Orrego, Margarita Castillo, Hugo Sáenz, Luis Álvaro Hernández, Scott Weichenthal, Jill Baumgartner, Néstor Y. Rojas

**Affiliations:** 1 Departamento de Salud Pública, Universidad Industrial de Santander, Carrera 32 29-31, Bucaramanga, Colombia; 2 Escuela de Ingeniería Civil, Industrial de Santander, Carrera 27 Calle 9 Ciudad Universitaria, Bucaramanga, Colombia; 3 Facultad Nacional de Salud Pública, Universidad de Antioquia, Calle 62 52-59, Medellín, Colombia; 4 Facultad de Ingeniería, Universidad Nacional de Colombia, Carrera 45 26-85 Edificio 401, Bogotá, Colombia; 5 Departamento de Ingeniería Civil y Ambiental, Universidad del Norte, Km 5 Vía Puerto Colombia, Barranquilla, Colombia; 6 Facultad de Ciencias de La Salud, Universidad Autónoma de Bucaramanga, Calle 157 15-55 El Bosque, Floridablanca, Colombia; 7 Escuela de Medicina, Universidad Pontificia Bolivariana, Calle 78B 72ª-159, Medellín, Colombia; 8 División de Investigación en Salud Pública, Instituto Nacional de Salud, Avenida Calle 26 51-20, Bogotá, Colombia; 9 Facultad de Ingenierías, Universidad San Buenaventura, Carrera 56C 51-110, Medellín, Colombia; 10 Área Metropolitana de Bucaramanga, Calle 89 Transveral Oriental Metropolitana, Bucaramanga, Colombia; 11 Corporación Para La Defensa de La Meseta de Bucaramanga, Carrera 23 37-63, Bucaramanga, Colombia; 12 Departamento Administrativo de Gestión del Medio Ambiente, Alcaldía de Santiago de Cali, Avenida 5AN 20-08, Cali, Colombia; 13 Área Metropolitana del Valle de Aburrá, Carrera 53 40ª-31, Medellín, Colombia; 14 EPA Barranquilla Verde, Carrera 60 72-19, Barranquilla, Colombia; 15 Secretaría Distrital de Ambiente, Alcaldía de Bogotá, Avenida Caracas 54-38, Bogotá, Colombia; 16 Department of Epidemiology, Biostatistics & Occupational Health, McGill University, 2001 McGill College Avenue, Montreal, Canada

**Keywords:** Air pollution, Fine particulate matter, Nitrogen dioxide, Land use regression models, Colombia

## Abstract

Rapidly urbanizing cities in Latin America experience high levels of air pollution which are known risk factors for population health. However, the estimates of long-term exposure to air pollution are scarce in the region. We developed intraurban land use regression (LUR) models to map long-term exposure to fine particulate matter (PM_2.5_) and nitrogen dioxide (NO_2_) in the five largest cities in Colombia. We conducted air pollution measurement campaigns using gravimetric PM_2.5_ and passive NO_2_ sensors for 2 weeks during both the dry and rainy seasons in 2021 in the cities of Barranquilla, Bucaramanga, Bogotá, Cali, and Medellín, and combined these data with geospatial and meteorological variables. Annual models were developed using multivariable spatial regression models. The city annual PM_2.5_ mean concentrations measured ranged between 12.32 and 15.99 µg/m^3^ while NO_2_ concentrations ranged between 24.92 and 49.15 µg/m^3^. The PM_2.5_ annual models explained 82% of the variance (*R*^2^) in Medellín, 77% in Bucaramanga, 73% in Barranquilla, 70% in Cali, and 44% in Bogotá. The NO_2_ models explained 65% of the variance in Bucaramanga, 57% in Medellín, 44% in Cali, 40% in Bogotá, and 30% in Barranquilla. Most of the predictor variables included in the models were a combination of specific land use characteristics and roadway variables. Cross-validation suggests that PM_2.5_ outperformed NO_2_ models. The developed models can be used as exposure estimate in epidemiological studies, as input in hybrid models to improve personal exposure assessment, and for policy evaluation.

**Supplementary Information:**

The online version contains supplementary material available at 10.1007/s11356-023-31306-w.

## Introduction

Air pollution is recognized as one of the leading environmental risk factors for population health (GBD 2019 Risk Factors Collaborators 2020). It is estimated that 99% of the world population is living in places where air pollution levels for fine particulate matter (PM_2.5_) exceed the current safe guideline level defined by the World Health Organization (WHO), and populations from low- and middle-income countries are exposed to the highest levels (World Health Organization 2021). In 2019, it was estimated that a total of 2.92 million deaths in females and 3.75 million deaths in males were attributable to ambient particulate matter and ozone air pollution. For Latin America and the Caribbean (LAC) region, and overall for low- and low-middle income countries, air pollution was the second most important risk factor (after malnutrition) that accounted for attributable disability-adjusted life-years (DALYs) rates over the past decade (GBD 2019 Risk Factors Collaborators 2020).

Particulate matter (PM_2.5_), nitrogen dioxide (NO_2_), and ozone (O_3_) are the ambient air pollutants most strongly associated with adverse health adverse effects in the short- and long-term (World Health Organization 2021). The health effects from long-term exposure to air pollution are tenfold higher than the short-term effects represented by daily variations (Pope 2007). For long-term exposure there is also evidence that there are large within-city contrasts and their effects are probably higher than the effects related to variations between cities (Crouse et al. 2015). Therefore, high-resolution spatial estimations of long-term exposure to air pollutants, particularly in urban setting, are critical for epidemiological research studying the association between air pollution and health and an important input for air quality management plans aimed to reduce air pollution adverse effects (Fann et al. 2011). There are different methods for estimating intraurban spatial variability of air pollutants. These methods include models based on proximity to monitoring stations, interpolation methods, land use regression models (LUR), and dispersion and chemical transport models combined with satellite remote sensing (Dijkema et al. 2011; Hoek 2017; Hoek et al. 2008; Michael Jerrett et al. 2005; van Donkelaar et al. 2021).

LUR models combined monitoring of air pollutants with the development of stochastic models using physical landscape characteristics, meteorology, and population as predictor variables (Hoek et al. 2008). LUR models use standard multivariable spatial regression techniques that have lower computational requirements compared with dispersion or chemical transport models and are relatively easy to implement using geographic information systems (GIS), which made them a method of preference in developing intraurban surfaces of air pollutant exposure (Hoek et al. 2008). LUR models have shown to have a high predictive value and to be a cost-effective method to estimate intraurban variations of air pollutants in different regions including North America, Europe, and Asia (Allen et al. 2011; Chen et al. 2013; de Hoogh et al. 2016; Eeftens et al. 2012; Gurung et al. 2017; Kashima et al. 2018; Lee et al. 2017; Stafoggia et al. 2022). Recently LUR has been used in these regions as input data for hybrid models combining dispersion models, satellite-based observations, land use, and surface monitoring data for PM_2.5_ and NO_2_ (Hoek 2017). Also, annual and monthly global estimates of ground level PM_2.5_ and NO_2_ have been developed, combining satellite remote sensing with the GEOS-Chem chemical transport model and calibration using ground-level observations (van Donkelaar et al. 2021). These models provide spatially fine resolutions at 0.01° × 0.01° and have shown to have a very good performance in North America and Europe but have very high uncertainty for tropical areas particularly in South America (Hoek 2017; van Donkelaar et al. 2021).

Despite LAC cities are growing rapidly and experiencing high levels of air pollution, the estimates of long-term exposure to air pollution are scarce in the region. In most cities, the ground-level measurements of atmospheric pollutants have poor consistency and coverage (Cunha-Zeri and Ometto 2021). Limitations include that traditional air quality stations require high financial funding in resource-limited countries which make them logistically prohibitive since it is not cost-effective. Consequently, given the limited resources of good air quality data, modeling emerges as a possible tool to derive management measures (Agudelo-Castañeda et al. 2023). However, high-resolution spatial estimations of long-term exposure to air pollutants are scarce in LAC and development of LUR models for some pollutants have been reported only for the cities of Mexico, Sao Paulo, Quito, and Medellín (Alvarez-Mendoza et al. 2019; Habbermann and Gouveia 2007; Londoño and Cañon 2015; Luminati et al. 2021; Son et al. 2018).

Colombia is located at the extreme north of South America with an estimated population of 52 million inhabitants (Departamento Nacional de Estadística (DANE) 2020b) distributed across 32 departments and 1122 municipalities. The national air quality surveillance network has operated since 1993 and currently includes 22 regional surveillance systems that are distributed in 77 municipalities of 19 departments. In 2021, the national monitoring network included 131 monitoring stations for PM_2.5_ and 57 for NO_2_ (Instituto de Hidrologia Meteorología y Estudios Ambientales-IDEAM 2022). Data from monitoring stations provide useful information for temporal daily variations of pollutants but provide limited information on the spatial variability of pollution especially in densely populated urban settings that concentrate 77% of the country’s population (Departamento Nacional de Estadística (DANE) 2020b). Data from monitoring surveillance systems have been used in epidemiological studies assessing the short-term effects of pollutant concentrations on mortality and morbidity in the largest cities in Colombia (Blanco-Becerra et al. 2014; Rodriguez-Villamizar et al. 2018). However, there is a need for estimations of long-term spatial variation of pollutants within cities. LUR models have provided high performance and less computational requirements compared to other methods for assessing long-term exposures to air pollutants. Therefore, our objective was to develop intraurban LUR models for PM_2.5_ and NO_2_ in the five largest cities in Colombia to estimate of long-term population exposure to air pollution for use in air quality health assessment and mitigation.

## Methods

### Study areas

**Fig. 1:**
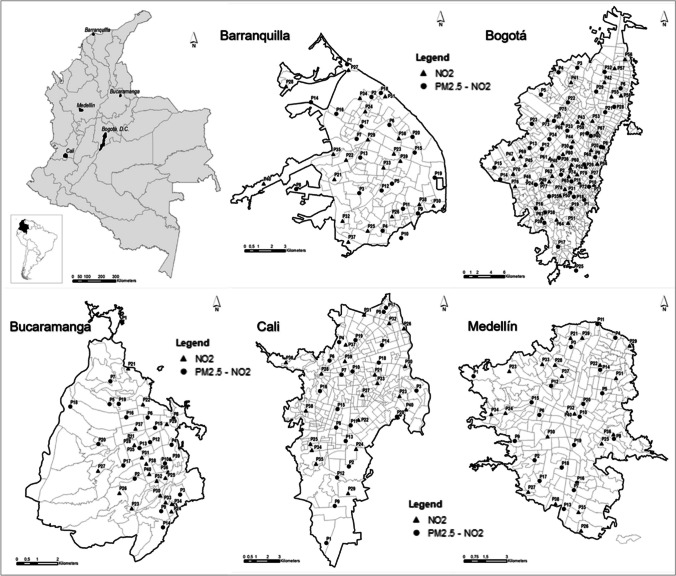
Study areas and monitoring location within cities in Colombia. Note: Circles represent monitoring sites for both pollutants, PM_2.5_ and NO_2_, and triangles represent monitoring sites for NO2 only

The study was conducted in the urban areas of the five largest cities in Colombia: Barranquilla, Bucaramanga, Bogotá, Cali, and Medellín (Fig. 1). The population varies across cities, Bogotá being the most populated city with an estimated population of 7,834,167 million inhabitants in 2021. The estimated total population during 2021 was 1,297,082 for Barranquilla; 614,269 for Bucaramanga; 2,264,748 for Cali; and 2,573,220 inhabitants for Medellín (Departamento Nacional de Estadística (DANE) 2020b). The altitude and average temperature also vary across cities, with Barranquilla being the warmer and closest to the sea level and Bogotá being the coldest and highest elevation. The physical characteristics of these cities are presented in Table S1 in Supplementary material. Similar to other capital cities in South America, the roadways networks in these cities are complex and dense, and both industrial and residential neighborhoods coexist.

### Air pollution measurement data

PM_2.5_ and NO_2_ concentrations were measured in the five cities for two consecutive weeks during both the dry and the rainy season in 2021. The selection of the dry and rainy seasons for each city was defined based on the total precipitation registered in local meteorological stations between 2010 and 2019. The driest months correspond to January to March while the months with higher precipitation were April to May for most cities. The details of the sampling period for each city are presented in Table S1 in Supplementary material.

For NO_2_, there were 80 sampling sites for Bogotá and 40 for the other cities; while for PM_2.5_, there were 40 sampling sites for Bogotá and 20 for the other cities. Figure 1 shows the location of sampling sites distributed across the urban area of the cities. The density of sampling sites in the urban areas for NO_2_ measurements (samplers per km^2^) was 2.3 for Barranquilla, 0.8 for Bucaramanga, 4.4 for Bogotá, 3.5 for Cali, and 3.6 for Medellín; the density of sampling for PM_2.5_ was twice these values as we used half the number of monitors. The selection of sampling sites was conducted with participation of the study team and experts from the environmental and health departments of each city. The criteria for selecting the monitoring sites included (1) the representation of traffic, residential, industrial or other areas within the cities, and (2) the heterogeneity in the characteristics of the selected sites (i.e., in terms of types of traffic, density of residential areas or particular areas for cities such as port or industrial areas). The sampling sites included one background urban site per city. The background site was located in the area of the city with the lowest concentrations of pollutants based on measurements, if they were available, or based on the experts’ knowledge of pollution within the city. In addition, sampling included 3–4 sites per city that were installed in the same location as monitoring stations from the local air quality network to facilitate instrument intercomparisons. For quality control, two blank filters were used for each city.

Measurement campaigns were simultaneously conducted across all sampling sites in each city for 2 weeks. Two trained teams of field staff were responsible for installing and uninstalling monitoring samplers across the study cities. We measured gravimetric PM_2.5_ using Ultrasonic Personal Aerosol Sampler (UPAS) samplers (V2.0 Access Sensor Technologies, Fort Collins, CO, USA) that were installed between 2.5 and 3 m above ground in all monitoring sites. The UPAS monitors have been widely used for measuring gravimetric PM_2.5_ in similar and higher pollution settings (Arku et al. 2020) and have shown good performance for collecting airborne PM for gravimetric analysis (Leith et al. 2020). We adapted an environmental enclosure to protect the device during outdoor sampling and added an external battery to increase the sampling time to 7 days at 25% duty cycle at a flow rate of 1 lpm. Each monitor was loaded with a 37-mm Teflon filter at the start of each measurement period. We replaced the UPAS and filters at each sampling site after 7 days to complete the 2 weeks monitoring period. Gravimetric analysis was conducted for all cities in a single laboratory certified for this competence (ISO/IEC 17025:1999) by the Instituto de Hidrología, Meteorología y Estudios Ambientales (IDEAM). Each filter and blank were weighted three times, and the average measurement was reported for each filter. The reported limit of quantification was 0.68 µg, and the limit of detection was 1.36 µg. The average PM_2.5_ concentration of the two weekly filters from the same site, and campaign was reported as the site concentration for statistical analysis.

For measuring NO_2_, we used passive diffusion Palmes Tubes (Gradko environmental, Hampshire, UK) that were installed for 2 weeks with a height of 2.5–3 m above ground in all monitoring sites. For quality control, an extra two blank tubes were deployed in each city. The processing of all tubes was conducted in the manufacturers’ laboratory, and concentration measurements were reported as the average of duplicate measurements. The reported limit of detection was 0.031 µg of NO_2_ in tubes. The installation, operation, and deinstallation of the PM_2.5_ and NO_2_ monitoring devices including refrigeration of samples were conducted by trained personnel following the manufacturer’s instructions.

### GIS predictor variables

Predictor variables were grouped into five categories: (1) land use (areas of different land uses); (2) population (including population counts and population density); (3) roads (including total length of roads and distance from sampling sites to arterial roads); (4) traffic (including estimated average speed and traffic volume); (5) physical geography (altitude); and (6) meteorology (including average temperature, precipitation, relative humidity, and wind direction). All predictor variables were created for circular buffers with radii of 100 m, 200 m, and 500 m and centered at the monitoring sites. These predictor variables were obtained from the intersection between buffers and GIS layers. In total, 78 independent variables were generated including variations of roadways variables. Maps were created using ESRI ArcGIS® 10.8.1 and ArcMap™ under license (ESRI® version, US). Table 1 provides the details of the predictor variables used to generate the LUR models.

**Table 1:** Land use regression predictor variables^a^

Category	Source	Year	Unit	Variable names
Land use	Land use plan for each city	2014	% of land use type (square meters)	Industrial-IND Residencial-RES Dotacional-DOT Central-CEN Commercial-COM Port-PORT Mixed-MIX
Population	National Census	2018	People per square meter	Total population-POB Population density-DEN
Roads	Open Street Maps	2020	Kilometers	Length by road type Trunk road-TRUNK Primary road-PRIM Secondary road-SEC Tertiary road-TER Local road-LOC Distance from site to road by road type DTRUNK DPRIM DSEC DTER DLOC
Traffic	Speed Distance Matrix API—Google Speed-density-flow functions	2021	Kilometers per hour Vehicles per hour	Traffic speed-VEL Traffic volume-VOL
Physical geography Altitude		2021	Meters above sea level (MASL)	Altitude-ALT
Meteorology	Monitoring Station	2021	Temperature °C Precipitation mm Relative humidity % Wind direction	Temperature TPROM Precipitation PPROM Humidity (%) HR Wind direction WD

^a^All predicted variables were created for buffers of 100 m, 200 m, and 500 m

Land use data were obtained from the local government’s planning office based on the most recent land use distribution available. Altitude was measured in sampling sites directly using an altimeter during the first deployment of monitoring devices. Population data and roads classification were obtained from the demographic and cartographic information of the census 2018 (Departamento Nacional de Estadística (DANE) 2020a). Meteorology data were obtained from meteorological monitoring stations from the local environmental authority including 16 stations in Bogotá, 22 stations in Medellín, and 8 stations in Cali. Precipitation and temperature raster surfaces were calculated using the Regnie model (Rauthe et al. 2013). Briefly, we used data from stations coupled with altitude from the digital terrain model (DTM) with 30 m resolution, the slope and land exposure (the direction or azimuth angle of the inclination of the slope) to calculate spatial precipitation and temperature mean values using a linear regression model. Barranquilla and Bucaramanga had less than four local meteorological stations that did not allow for a valid spatial estimation, and therefore, meteorological data was not included in LUR models for these two cities.

Traffic predictor variables were measured and estimated for the project. The traffic speed measurements were obtained during the same monitoring campaigns periods by using a cloud-based data method that included data pre-processing, speed computation, and output data formatting. During the pre-processing, the street network vector data from Openstreetmap was edited to match the same network used by the Google Maps platform. Then, the network streets were split into 100-m links considering the road intersections setup. Then, the speed was computed for those links using their length and travel time. Travel times at the link level were obtained from the Google Maps platform using the distance matrix API service, which provides predicted values at the time the service was used. Finally, the speed of each link was added to its attributes set, and the whole collection of links was used to create a GIS layer using Python scripts.

To estimate traffic volumes, we used speed-density-flow functions, which describe the relationships between traffic speed, density, and flow rate on a road segment. These functions were obtained and used to estimate traffic conditions (National Research Council 2010). We computed speed-density-flow functions for urban traffic for Bogota (73 road segments) and Medellín (199 road segments) using data from sensors and traffic cameras provided by the transportation authorities. We computed and validated the functions for three different traffic regimes: interrupted, semi-interrupted, and uninterrupted flow. We tested six theoretical functional forms (Greenshields, Drew, Pipes, May&Keller, Greenberg, and Underwood Model) (Gaddam and Rao 2019) by using random sampling with replacement. The best model was selected based on the root mean square error (RMSE). The resulting functional forms were then used to estimate traffic volumes in the road network of Barranquilla, Cali, and Bucaramanga, taking into account the traffic regimes, and the number of lanes in each road segment.

### Statistical analysis

We averaged pollutants’ concentrations measured during both sampling campaigns to obtain annual means for each city. The comparison of measurements of the PM_2.5_ sampling device with local monitoring stations was conducted for 13 monitoring stations with data available (2 in Barranquilla, 4 in Bogotá, 4 in Cali, and 3 in Medellín). Comparison of concentrations was evaluated using Bland and Altman agreement coefficients and graphs (Bland and Altman 1986). The average annual measurements across the monitoring sites were also compared to the average annual estimation measurements from the real-time local monitoring stations in the cities.

We developed LUR models to estimate intraurban spatial variation of PM_2.5_ and NO_2_ within the five cities. We used multivariable spatial regression models that allow local estimations of a dependent variable *Z*, by implementing the ordinary least squares (OLS) method, in the presence of possible explanatory variables (${Z}_{j}$) at the same point $\left({x}_{i},{y}_{i}\right)$ represented by the following equation (Londoño, 2018; Maantay and McLafferty 2011):

$$\begin{array}{cc}Z\left({x}_{i},{y}_{i}\right)={\beta }_{0}+{\sum }_{j=1}^{n}{\beta }_{i}{Z}_{j}\left({x}_{i},{y}_{i}\right)+{\varepsilon }_{j},& {\varepsilon }_{j}\sim N\left(0,{v}^{2}\right)\end{array}$$

To represent the spatial dependency structure between the features being analyzed, the best combination of explanatory variables must be determined. In a first step, we removed highly correlated variables (> 0.7) and those variables in which zero values account for more than 90% of the sampling sites. Then, all the predictors are included in the model assessing their statistical significance (*p* value < 0.05) and the sign for their coefficient (β*i*) (observing their agreement with the expected theoretical direction of effect). In addition, the selected variables must adequately specify the regression model, by evaluating the specification criteria of the OLS method. We estimated the adjusted *R*-squared to assess the performance of the models and the variance inflation factor to determine multicollinearity. All models were built with a combination of all the buffer variables (Eeftens et al. 2012; Van Nunen et al. 2017).

We performed a geographically weighted regression (GWR) with the selected equation to examine the spatial heterogeneity of the relationship between air pollutants and other spatial variables and to estimate the multiple regression model parameters. Then, we created a regular point mesh with cells spaced by 200 m over the cities’ surface, where the formula obtained by each annual regression model was applied, in order to predict air pollutant levels for each point. Then, a spatial interpolation method (spline) was applied to obtain the concentration surface of the pollutant in the study area. Finally, we performed a leave-one-out cross validation (Eeftens et al. 2012; Wang et al. 2016) for each LUR model in each city and compared the set of predicted values against the observed ones. Then, the cross-validated square error and *R*^2^ were calculated for each model. The cross-validation was conducted using the “loocv” command in Stata® version 13 (Stata Corporation).

## Results

### Pollutants’ concentrations at sampling locations

There were 116 PM_2.5_ sampling sites with valid measurements for both monitoring campaigns used for the estimation of the annual average concentrations. Three sites in Cali, four sites in Bogotá, and one in Medellin were excluded because they contribute only one successful measurement. The mean PM_2.5_ concentrations during the dry season were slightly higher compared to the rainy season (see supplementary material Table S1). The annual PM_2.5_ mean concentration and range in sampling sites were 16.12 µg/m^3^ (7.42–22.22) for Medellín, 15.90 µg/m^3^ (3.64–35.30) for Barranquilla, 15.79 µg/m^3^ (4.86–32.69) for Cali, 13.89 µg/m^3^ (4.39–25.52) for Bogotá, and 12.93 µg/m^3^ (4.90–32.23) for Bucaramanga.

For NO_2_ sampling, 17 out of the 240 tubes deployed were removed due to vandalism or invalid measurements, leaving 223 observations for the analyses. The mean NO_2_ concentrations during the dry season were slightly higher than those in the rainy season (see supplementary material Table S1). The annual NO_2_ mean concentration and range in sampling sites were 49.09 µg/m^3^ (32.38–68.31) for Medellín, 34.92 µg/m^3^ (12.56–64.67) for Bucaramanga, 39.12 µg/m^3^ (13.52–69.89) for Cali, 34.63 µg/m^3^ (5.09–52.19) for Bogotá, and 24.92 µ/m^3^ (7.38–51.81) for Barranquilla.

The average of the differences in PM_2.5_ concentrations measured using the UPAS and those reported during the same sampling period by local monitoring stations was − 1.5 µg/m^3^ (95%CI − 6.8 to 3.9) during the dry season campaign (11 monitoring stations) and − 0.05 µg/m^3^ (95% CI − 11.5 to 11.4) during the rainy season campaign (13 monitoring stations). During the dry season campaign, higher differences were observed for two local monitoring stations, one in Medellín and one in Cali. During the rainy season campaign, higher differences were observed for the three local monitoring stations from Medellín. Figure S1 shows the levels of agreement for PM_2.5_ measurements during the two monitoring campaigns. There was only one monitoring station in downtown Medellín with valid NO_2_ data for comparison of measurements obtained from Palmes tubes and local monitors. For this site-station pair, the differences was 5.71 and 2.59 µg/m^3^ during the dry and rainy season, respectively. In Bogotá during the second campaign (rainy season), there were four sites with valid paired measurements whose average difference was 6.70 µg/m^3^, which was highly influenced by the discrepancy observed in one particular station located at Carrera 7a (excluding this station the average of the difference was 2.86 µg/m^3^). The comparison of the PM_2.5_ average campaign’s measurements from monitoring sites with the average annual measurements from monitoring stations during 2021 resulted in differences of − 0.84 µg/m^3^ for Bucaramanga, − 1.1 µg/m^3^ for Medellín, − 1.7 µg/m^3^ for Bogotá, 1.4 µg/m^3^ for Cali, and 1.7 µg/m^3^ for Barranquilla. For NO_2_, the difference between passive samplers and monitoring stations in Bogotá was 5.6 µg/m^3^.

### LUR models

**Fig. 2:**
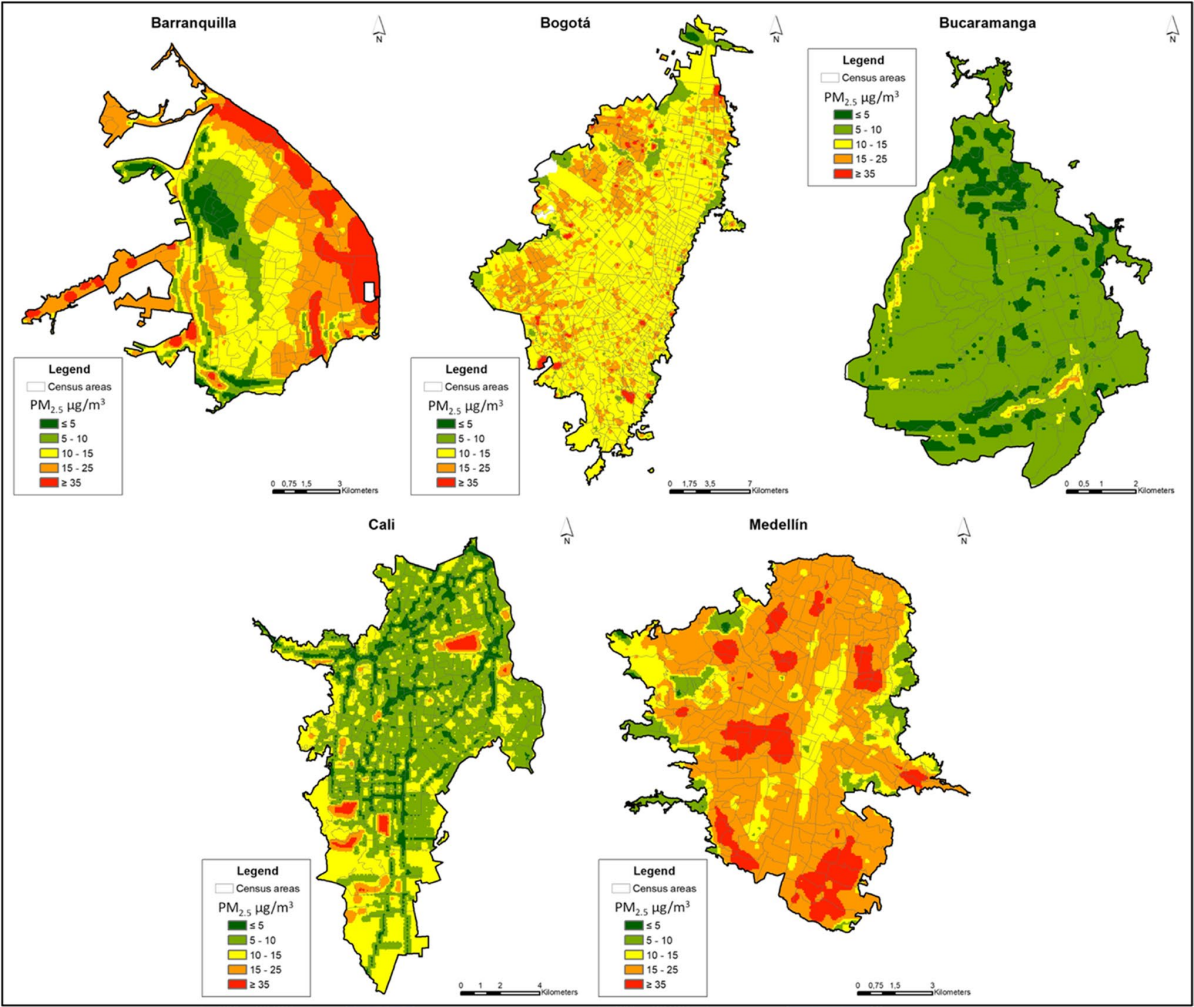
Annual predicted concentrations for PM_2.5_ in five cities in Colombia, 2021

The final LUR models selected for the cities explained higher variability for PM_2.5_ compared with NO_2_ (Tables 2 and 3, respectively). The models for PM_2.5_ explained between 44% (Bogotá) and 82% (Medellín) of pollutant’s spatial variability within cities. Most models showed a RMSE of approximately 1.5 µg/m^3^ except for Barranquilla where the error was approximately 4 µg/m^3^. The contrasts between PM_2.5_ measured and predicted concentrations at monitoring sites for all cities are presented in Supplementary material Figure S2. Most of the predictor variables included in the PM_2.5_ LUR models were related to specific types of land uses and roadways’ attributes with predominance of 200 and 500 m buffers. In Bucaramanga, the LUR model only included roadways variables while Medellín was the only city where the model included a meteorological variable (see Table 2). There was no evidence of multicollinearity in the LUR models for both pollutants as the VIF values were all below 2.1. The maps of the predicted concentrations for PM_2.5_ in the urban areas of the five cities are presented in Fig. 2.

**Table 2:** Description of developed LUR models for PM_2.5_ in five cities in Colombia, 2021

City	LUR model	No. sites	Model *R*^2^	RMSE	VIF	*R*^2^ cross validation
Barranquilla	PM_2.5_ = 19.83344–0.1489524*ALT–0.0230902*DTRON500 + 44.43591*IND200 + 21.93109*CEN500 + 23.10317*PORT500	20	0.73	3.98	1.90	0.54
Bogotá	Ln (PM_2.5_) = 2.4713 + 3.1439*DEN100 + 1.8045*IND200–0.8418*RES500	40	0.44	1.39	1.63	0.38
Bucaramanga	Ln (PM_2.5_) = 2.199057 + 0.0014062 ∗ SEC100 + 0.0000327 ∗ LOC500–0.0012659 ∗ DPRIM500 + 0.0215501 ∗ VEL100–0.000242 ∗ VOL200	20	0.77	1.23	1.78	0.46
Cali	Ln (PM_2.5_) = 2.3387 + 0.00001 ∗ DOT200 + 1.0713 ∗ PRIM200 + 0.5943 ∗ SEC200–0.0004 ∗ VOL100	17	0.70	1.28	2.06	0.51
Medellín	PM_2.5_ = 13.77207–1.357455 ∗ PPROM–5.589831 ∗ DOT100 + 2.269679 ∗ DEN200 + 70.23039 ∗ MIX500 + 0.0043842 ∗ VOL500	19	0.82	1.71	1.48	0.79

*RMSE* Root mean square error, *VIF* variance inflation factor, *ALT* altitude, *CEN*, central land use, *DEN* population density; *DOT* Dotacional land use; *DPRIM* distance to primary roadway; *DTRON* distance to trunk roadway; *IND* industrial land use; *LOC* length local roadways; *MIX* mixed land use; *PORT* Port land use; *PPROM* precipitation average; *PRIM* length primary roadways; *RES* residential land use; *SEC* length secondary roadways; *VEL* vehicular speed; *VOL* vehicular volume. Numbers correspond to buffers of 100 m, 200 m, and 500 m

**Table 3:** Description of developed LUR models NO_2_ in five cities in Colombia, 2021

City	LUR model	No. sites	Model *R*^2^	RMSE	VIF	*R*^2^ cross validation
Barranquilla	NO_2_ = 12.89591 + 25.45936 ∗ PRIM100–0.1583713 ∗ VEL100 + 0.0061518 ∗ VOL500	36	0.30	8.02	1.28	0.19
Bogotá	Ln (NO_2_) = 2.8714 + 0.0001*PRIM500 + 0.0058*VEL100 + 0.2599*WPROM	73	0.40	1.26	1.19	0.34
Bucaramanga	NO_2_ = 13.00243 + 291.5302 ∗ DEN100–0.0013283 ∗ POB500 + 0.0025503 ∗ TER500 + 0.0020514 ∗ LOC500 + 0.0057464 ∗ VOL100	40	0.65	8.08	1.66	0.55
Cali	Ln (NO_2_) = 3.47834312 + 0.49126931*PRIM200 + 0.39823891 ∗ SEC200 + 0.36505469 ∗ TER200–0.01475995 ∗ VEL200	40	0.44	1.28	1.56	0.36
Medellín	NO_2_ = 46.06516–3.625967 ∗ PPROM–0.0299678 ∗ DSEC200 + 0.0225605 ∗ VOL500	34	0.57	5.53	1.06	0.45

*RMSE* Root mean square error, *VIF* variance inflation factor, *DEN* population density, *DOT* Dotacional land use, *DSEC* distance to secondary roadway; *LOC* length local roadways; *POB* population size; *PPROM* precipitation average; *PRIM* length primary roadways; *SEC* length secondary roadways; *TER* length tertiary roadways; *VEL* vehicular speed; *VOL* vehicular volume, *WPROM* wind speed (mean). Numbers correspond to buffers of 100 m, 200 m, and 500 m

**Fig. 3:**
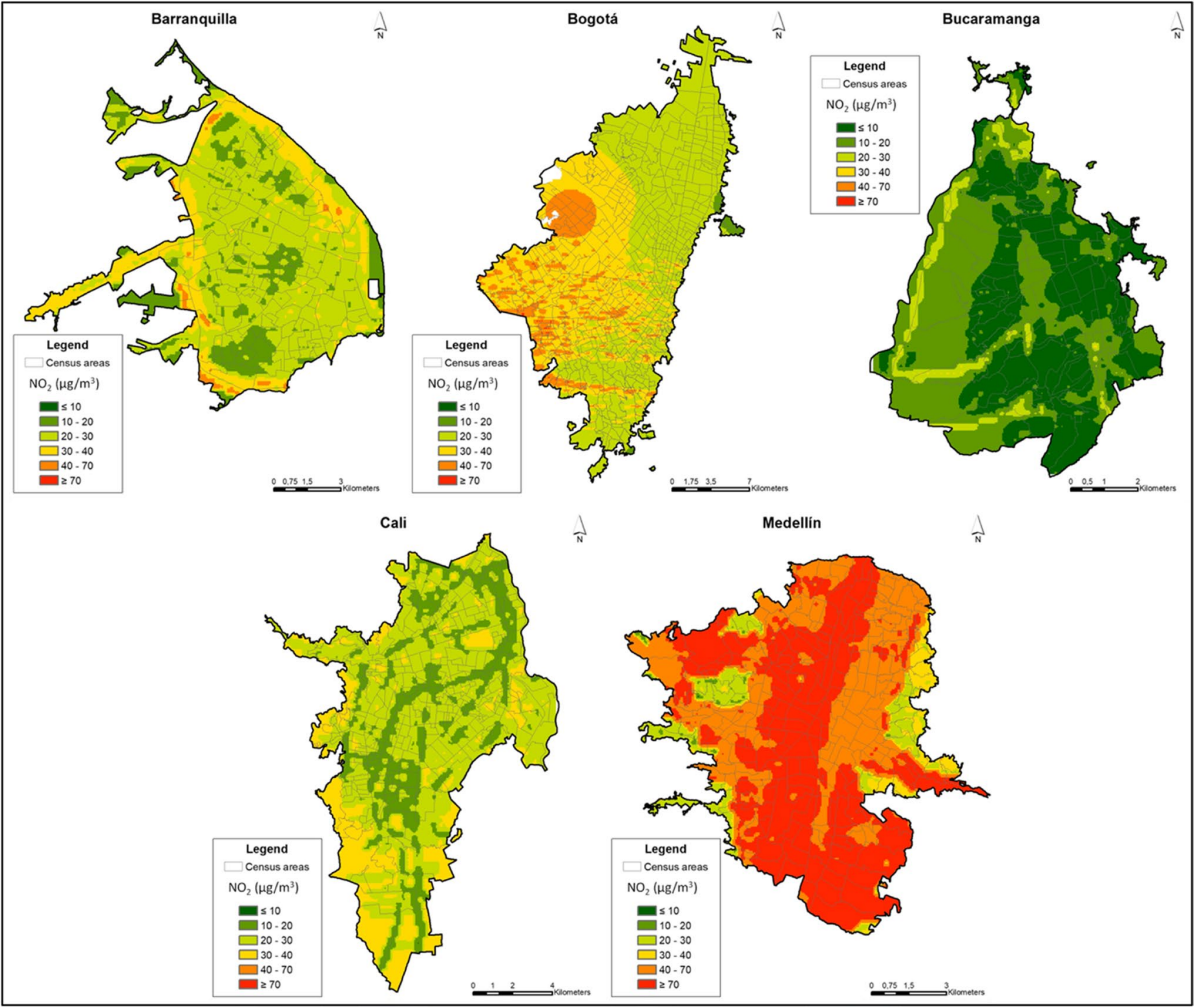
Annual predicted concentrations for NO_2_ in five cities in Colombia, 2021

The final selected models for NO_2_ explained between 30% (Barranquilla) and 65% (Bucaramanga) of the pollutant’s spatial variability within cities. Most cities models showed a RMSE around 6 to 8 µg/m^3^ except for Cali where the error was close to 1.5 µg/m^3^. The measured values versus the predicted values of the models in the monitoring sites for NO_2_ in all cities are presented in Supplementary material Figures S3. As expected, most of the predictor variables included in the NO_2_ LUR models were a combination of roadways variables with different buffers. In Bucaramanga, the LUR model included population variables and, in Medellín, one meteorological variable (see Table 2). There was no collinearity in the LUR models for both pollutants as the VIF values were all below 1.7. The maps of the predicted concentrations for NO_2_ in the urban areas of the five cities are presented in Fig. 3.

### Cross validation

Overall, the leave-one-out cross-validation *R*^2^s showed good stability, particularly for PM_2.5_. For PM_2.5_, the difference between the model *R*^2^ and the validation *R*^2^ was 19% for Barranquilla, 31% for Bucaramanga, 6% for Bogotá, 19% for Cali, and 3% for Medellín. For NO_2_, the difference between the model *R*^2^ and the validation *R*^2^ was 11% for Barranquilla, 10% for Bucaramanga, 6% for Bogotá, 8% for Cali, and 12% for Medellín. Validation *R*^2^s are presented in Tables 2 and 3 for PM_2.5_ and NO_2_, respectively.

## Discussion

This is the first study to develop LUR models for multiple cities in a Latin American country, providing small-area estimations of air pollutants for use in health risk assessments, epidemiological studies of long-term exposure to air pollution, and mitigation evaluation. The development of LUR models to estimate concentrations for PM_2.5_ and NO_2_ in five of the largest Colombian cities showed moderate to high explained variance, respectively. Generally, the models showed higher explained variance of PM_2.5_ compared with NO_2_. Among the cities, the lowest explained variance was obtained for Bogotá, while the highest was recorded for Medellín and Bucaramanga.

The LUR models for PM_2.5_ showed relatively small errors of the predicted concentrations (RMSE < 1.7 µg/m^3^) in the cities, except for Barranquilla. Moreover, the performance of the LUR models developed for PM_2.5_ was higher than that reported in previous studies in Colombia. Previous LUR models were available only for PM_10_ and PM_2.5_ in the city of Medellín with an explained variability of 79% for PM_10_ (Londoño and Cañon 2015) and monthly variations between 26 and 79% for PM_2.5_ (Grisales 2020), using data from 2007 and 2018, respectively. Our selected LUR model for PM_2.5_ in Medellín explained 82% of the variability, the highest of the five cities, using a combination of meteorological, land use, population density, and traffic volume variables. The high performance of the LUR models for PM_2.5_ in Medellín compared to other cities might be explained by the wide range of estimated concentrations in the city and the influence of the topography and meteorology in the Valley of Aburrá where Medellín is located, as well as the important contribution of vehicular emissions to local concentrations as have been described in studies of PM_2.5_ characterization in the city (Area Metropolitana del Valle de Aburrá and Politecnico Colombiano Jaime Isaza Cadavid 2021). In contrast, the low performance of the LUR models for PM_2.5_ in Bogotá compared to other cities might be explained partially by the lower contribution of vehicular emissions and the increased contribution of enriched fugitive dust (resuspension of crustal material and soil dust) and secondary PM (Ramírez et al. 2018). A similar profile has also been documented for Barranquilla with an important contribution of ocean aerosols (Nuñez Blanco 2019), secondary organic aerosols and the effect of exposed land resuspension and road dust (Gómez-Plata et al. 2022), which was represented in the developed LUR model for this city. Additional unexplained variability in PM_2.5_ concentrations in the cities might be related to regional wildfires contributions which have been substantial in northern South America and particularly in Bogotá (Ballesteros-González et al. 2020) (Casallas et al. 2022).

The variation in explained variability reported for the Colombian cities is comparable to that of PM_2.5_ in other Latin American and European countries. In Ecuador, Alvarez et al. (Alvarez-Mendoza et al. 2019) developed LUR models for PM_10_ using remote sensing data, and the models showed an explained variability of 68% at its highest. Sangrador et al. (2008) developed LUR models for PM_2.5_ during the rainy season in 2003 for Mexico City, which showed an explained variability of 60%. Later, Son et al. (2018) developed LUR models for the same city for different temporal scales, and the best explained variability for monthly PM_2.5_ models was 76%. In Europe, the ESCAPE project developed LUR models for PM_2.5_ in 20 study areas, where the explained variability varied from 35% in Manchester, UK, to 89% in Paris, France (Eeftens et al. 2012).

As expected, the best predictor variables in our LUR models for NO_2_ were road and traffic variables. However, the performance of the LUR models developed for NO_2_, however, was lower than that for PM_2.5_ and the reported from previous studies in other countries. In Sao Paulo, an annual LUR developed for NO_2_ explained 66% of the variability in urban concentrations, with variations for summer (75%) and winter (52%) seasons (Luminati et al. 2021). For the Western European countries, Vienneau et al. (2013) developed LUR models for NO_2_ with and without satellite-based NO_2_ and obtained explained variability between 48 and 58% without satellite-based NO_2_ and a modest additional improvement of 5% when adding satellite-based data. In our models for NO_2_, despite including different variables and metrics of traffic and roads, the models could not capture a higher variability in concentrations, which suggests secondary reactions might be an important source of NO_2_ in the cities. Although our NO_2_ LUR explained less variability compared to other reported models in cities, the LUR models explain more variability than simple road proximity metrics or interpolation methods based on data from monitoring stations and similar variability than dispersion models, which have been demonstrated in previous studies assessing exposure assessment for epidemiological studies (Allen et al. 2011; de Hoogh et al. 2014; Jerrett et al. 2007).

The LUR models have been used in exposure assessment and health research related to long-term exposure to air pollutants. By incorporating data on local sources of pollution, such as traffic or industrial activity, these models can provide more accurate and precise exposure estimates than traditional monitoring methods (Hoek et al. 2008). This is particularly important for assessing the health effects of chronic exposure to air pollution, which has been linked to a range of adverse health outcomes, including respiratory and cardiovascular disease, cancer, and neurological disorders (Chen et al. 2013; Herting et al. 2019; Knibbs et al. 2018; Lamichhane et al. 2017; Stafoggia et al. 2022). LUR models can also identify areas of high pollution levels and vulnerable populations, helping to inform policy and intervention strategies to reduce exposure and improve public health (Vienneau et al. 2013).

Alternative methods for estimating surface concentrations of air pollutants have been developed recently using satellite-based models and models using mobile air pollutant measurements. A study conducted at the municipality level in Colombia compared air quality models based on satellite measurements for PM_2.5_ between 2014 and 2019. It showed that the Copernicus Atmospheric Monitoring Service Reanalysis (CAMRA) and the Atmospheric Composition Analysis Group (ACAG) models had a low correlation and tended to overestimated surface concentrations when both models were compared to surface data from 28 cities in 2019. However, ACAG outperformed CAMSRA in terms of mean bias of the model and the spatial representation of the highest concentrations (Rodriguez-Villamizar et al. 2022). Using a mobile monitoring campaign in the city of Bucaramanga in 2019, estimations of within-city spatial variations in ultrafine particle and black carbon concentrations were predicted using a combination of LUR and convolutional neural networks trained using satellite and street-level images, showing the improvement of prediction when using a hybrid approach (Lloyd et al. 2021). Following this hybrid approach, our locally developed LUR models can be further used to develop hybrid models with satellite or mobile data and produce better spatially calibrated models for estimating long-term exposure for PM_2.5_ and NO_2_ in the main cities in Colombia and explore their potential transferability across cities.

There are some strengths in our study that are worth mentioning. First, there was a good agreement between PM_2.5_ measurements made with UPAS compared to the concentrations reported by the local monitoring stations in the cities. For NO_2_, there were few monitoring sites to conduct a valid comparison in all cities, but data from local government stations in Bogotá had a good agreement with concentrations reported from measurements with the Palmes tubes. Second, we followed the same standardized procedure for conducting measuring pollutants during the two campaigns in each city and the simultaneous measurement within cities avoid the potential error related to using measures in different time scales. Third, we included basic predictor variables for developing LUR models in the cities (land use, roads, traffic, population, and meteorology) available in the cities in Colombia and might be used further to developed multi-city models as those developed for Europe (Wang et al. 2014).

One limitation of the LUR models developed for the cities is the limited number of sampling sites which was 20 for PM_2.5_ and 40 for NO_2_, except for Bogotá which doubled the number. These numbers are below the lower range of recommended monitoring sites (between 80 and 100) for modeling intraurban variations in complex urban settings using LUR (Basagaña et al. 2012). As a result, the models developed using many predictors might have resulted in more unstable performance as was observed in the cross-validation. A second limitation of this study is the absence of valid traffic data for the cities during the campaign measurement, which has shown to improve the LUR model performance, particularly for NO_2_ (Beelen et al. 2013). To overcome this limitation, we measured traffic speed derived from satellite instruments and used previously available traffic count data for the largest cities to calculate density functions which were then transferred to the other cities to estimated traffic density. Despite the density functions in the cities seemed to reflect the traffic patterns in the cities and were included as significant predictive variables, their inclusion did not help to explain a higher variability in the models for NO_2_. Third, we did not include meteorological variables in the development of LUR models for the cities of Bucaramanga and Barranquilla due to limited number of meteorological stations and data to produce a valid estimated surface. Although the models’ performance for PM_2.5_ were good particularly for Bucaramanga, including meteorological variables might have increased the models’ performance as they have been reported as important predictors for intraurban variations in other countries (Cheewinsiriwat et al. 2022; Olvera Alvarez et al. 2018). Another limitation of our study is that we did not include local emission sources and regional sources (such as forest fires) in the prediction models. These variables have shown to influence the concentration of particles in the cities (Casallas et al. 2022). Moreover, street NO_2_ levels may vary in building density or location, influencing their dispersion. Also, some atmospheric chemical reactions may reduce or transform NO_2_ concentrations. In urban areas, NO_2_ emitted mostly from traffic within a radius of 100–300 m showed a correlation, although the high reactivity of NO_2_ and rapid photodissociation may transform this pollutant in a reduced period (Agudelo-castañeda et al. 2020).

## Conclusion

In this study, we developed LUR models to predict PM_2.5_ and NO_2_ exposure in five main cities in Colombia. The LUR models showed a large intraurban variability of pollutant concentrations in all cities. The annual models for PM_2.5_ outperformed the models for NO_2_ and provided robust models that can be used in epidemiological studies, particularly cohort studies, assessing the effects of long-term air pollution on human health. The newly developed LUR models might be further used to create hybrid models in combination with other data sources to improve personal exposure assessment.

### Supplementary Information

Below is the link to the electronic supplementary material.

Supplementary file1 (DOCX 661 KB)

## Data Availability

The datasets used and/or analyzed during the current study are available from the corresponding author on reasonable request.
