# Supplementary material for: Intra-urban variability of long-term exposure to PM2.5 and NO2 in five cities in Colombia
Source: Environ Sci Pollut Res Int. 2023 Dec 12;31(2):3207–21. doi: 10.1007/s11356-023-31306-w (PMC10791881; doi:10.1007/s11356-023-31306-w)
Supplement: Supplementary file 1 — Supplementary file1 (DOCX 661 KB) [file 11356_2023_31306_MOESM1_ESM.docx]

**Intra-urban variability of long-term exposure to PM_2.5_ and NO_2_ in five cities in Colombia**

**Supplementary material**

Laura A. Rodriguez-Villamizar^1^, Yurley Rojas^2^, Sara Grisales^3^, Sonia C. Mangones^4^, Jhon J. Cáceres^2^, Dayana M. Agudelo-Castañeda^5^, Víctor Herrera^1,6^, Diana Marín^7^, Juan G. Piñeros Jiménez^3^, Luis C. Belalcázar-Ceron^4^, Oscar Alberto Rojas-Sánchez^8^, Jonathan Ochoa Villegas^9^, Leandro López^1^, Oscar Mauricio Rojas^10^, María C. Vicini^11^, Wilson Salas^12^, Ana Zuleima Orrego^13^, Margarita Castillo^14^, Hugo Sáenz^15^, Luis Álvaro Hernández^15^, Scott Weichenthal^16^, Jill Baumgartner^16^, Néstor Y. Rojas^4^

^1^ Departamento de Salud Pública, Universidad Industrial de Santander, Bucaramanga, Colombia; e-mail: laurovi@uis.edu.co; vicmaher@uis.edu.co

^2^ Escuela de Ingeniería Civil, Industrial de Santander, Bucaramanga, Colombia; e-mail: yurleyrg18@gmail.com, jcaceres@uis.edu.co

^3^ Facultad Nacional de Salud Pública, Universidad de Antioquia, Medellín, Colombia; e-mail: sara.grisales@udea.edu.co; juan.pineros@udea.edu.co

^4^ Facultad de Ingeniería, Universidad Nacional de Colombia, Bogotá, Colombia; e-mail: scmangonesm@unal.edu.co; lcbelalcazarc@unal.edu.co; nyrojasr@unal.edu.co

^5^ Departamento de Ingeniería Civil y Ambiental, Universidad del Norte, Barranquilla, Colombia; e-mail: mdagudelo@uninorte.edu.co

^6^ Facultad de Ciencias de la Salud, Universidad Autónoma de Bucaramanga, Bucaramanga, Colombia

^7^ Escuela de Medicina, Universidad Pontificia Bolivariana, Medellín, Colombia; e-mail: dianamarcela.marin@upb.edu.co

^8^ División de Investigación en Salud Pública, Instituto Nacional de Salud, Bogotá, Colombia; e-mail: orojas@ins.gov.co

^9^ Facultad de Ingenierías, Universidad San Buenaventura, Medellín, Colombia; e-mail: jonathan.ochoa@usbmed.edu.co

^10^ Área Metropolitana de Bucaramanga, Bucaramanga, Colombia; e-mail: oscar.rojas@amb.gov.co

^11^ Corporación para la defensa de la meseta de Bucaramanga, Bucaramanga, Colombia; e-mail: maría.vicini@cdmb.gov.co

^12^ Departamento Administrativo de Gestión del Medio Ambiente, Alcaldía de Santiago de Cali; e-mail: calidadairedagma@cali.gov.co

^13^ Área Metropolitana del Valle de Aburrá, Medellín, Colombia; e-mail: ana.orrego@metropol.gov.co

^14^ EPA Barranquilla Verde, Barranquilla, Colombia; e-mail: margarita.castillo@barranquillaverde.gov.co

^15^ Secretaría Distrital de Ambiente, Alcaldía de Bogotá, Bogotá, Colombia; e-mail: hugo.saenz@ambientebogota.gov.co; alvaro.hernandez@ambientebogota.gov.co

16 Department of Epidemiology, Biostatistics & Occupational Health, McGill University, Montreal, Canada; e-mail: scottandrew.weichenthal@mcgill.ca; jill.baumgartner@mcgill.ca

Table S1. Characteristics of the cities and sampling campaigns

| **Characteristics** | **Barranquilla** | **Bogotá** | **Bucaramanga** | **Cali** | **Medellín** |
| --- | --- | --- | --- | --- | --- |
| Population 2021^1^ | 1,297,082 | 7,834,167 | 614,269 | 2,264,748 | 2,573,220 |
| Urban extension coverage (Km^2^)^2^ | 92 | 349 | 31 | 139 | 142 |
| Altitude (masl) | 24 | 2,640 | 950 | 926 | 1,405 |
| Temperature ºC (anual mean) | 26.61 | 14.84 | 22.70 | 23.32 | 22.73 |
| Dry season sampling period | PM_2.5_ and NO_2_: April 19 – May 5 | PM_2.5_: March 17 – 29 (week 1) April 5-14 (week 2)^3^  NO_2_: April 20 -May 10 | PM_2.5_: Februray 24 – March 15. NO_2_: April 19-May 7 | PM_2.5_ and NO_2_: August 5-20 | PM_2.5_: August 20 -September 3 NO_2_: August 27-September 13 |
| PM_2.5_ mean concentration during dry season (μg/m^3^) | 16.71 | 17.33 | 13.85 | 15.38 | 16.21 |
| NO_2_ mean concentration during dry season (μg/m^3^) | 20.09 | 35.26 | 32.44 | 38.10 | 49.15 |
| Rainy season sampling period | PM_2.5_ and NO_2_: September 10 -24 | PM_2.5_ and NO_2_: July 14 - 30 | PM_2.5_ and NO_2_: June 15-30 | PM_2.5_ and NO_2_: September 30 - October 15 | PM_2.5_ and NO_2_: April 28 - Mayo 14 |
| PM_2.5_ mean concentration during rainy season (μg/m^3^) | 15.11 | 10.43 | 12.02 | 16.20 | 15.79 |
| NO_2_ mean concentration during rainy season (μg/m^3^) | 29.97 | 34.00 | 37.40 | 40.14 | 54.49 |

1 Estimations of population based on national census 2018. Source: DANE Colombia

2 Area delimited manually including the city urban area using Google Earth ®.

3 The first sampling campaign in cities, particularly in Bogotá, was delayed because of social protests. In Bogotá the first sampling campaign excluded the holy week as these days usually correspond to holiday season.

Figure S1. Agreement between PM2.5 measurements from sampling low-cost devices and local monitoring stations in cities

| 1. Dry season campaign (C1)   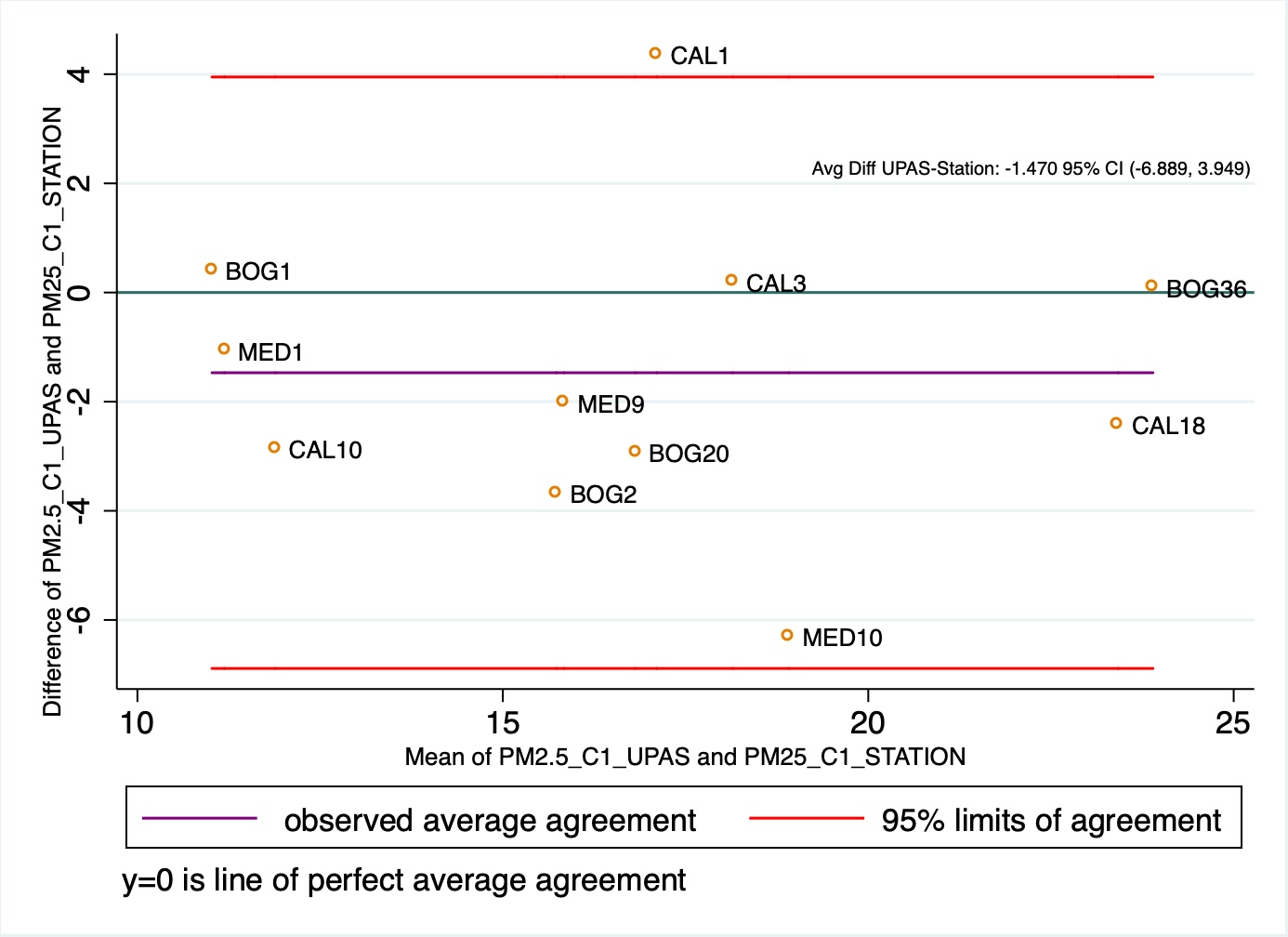 |
| --- |
| 1. Rainy season campaign (C2)   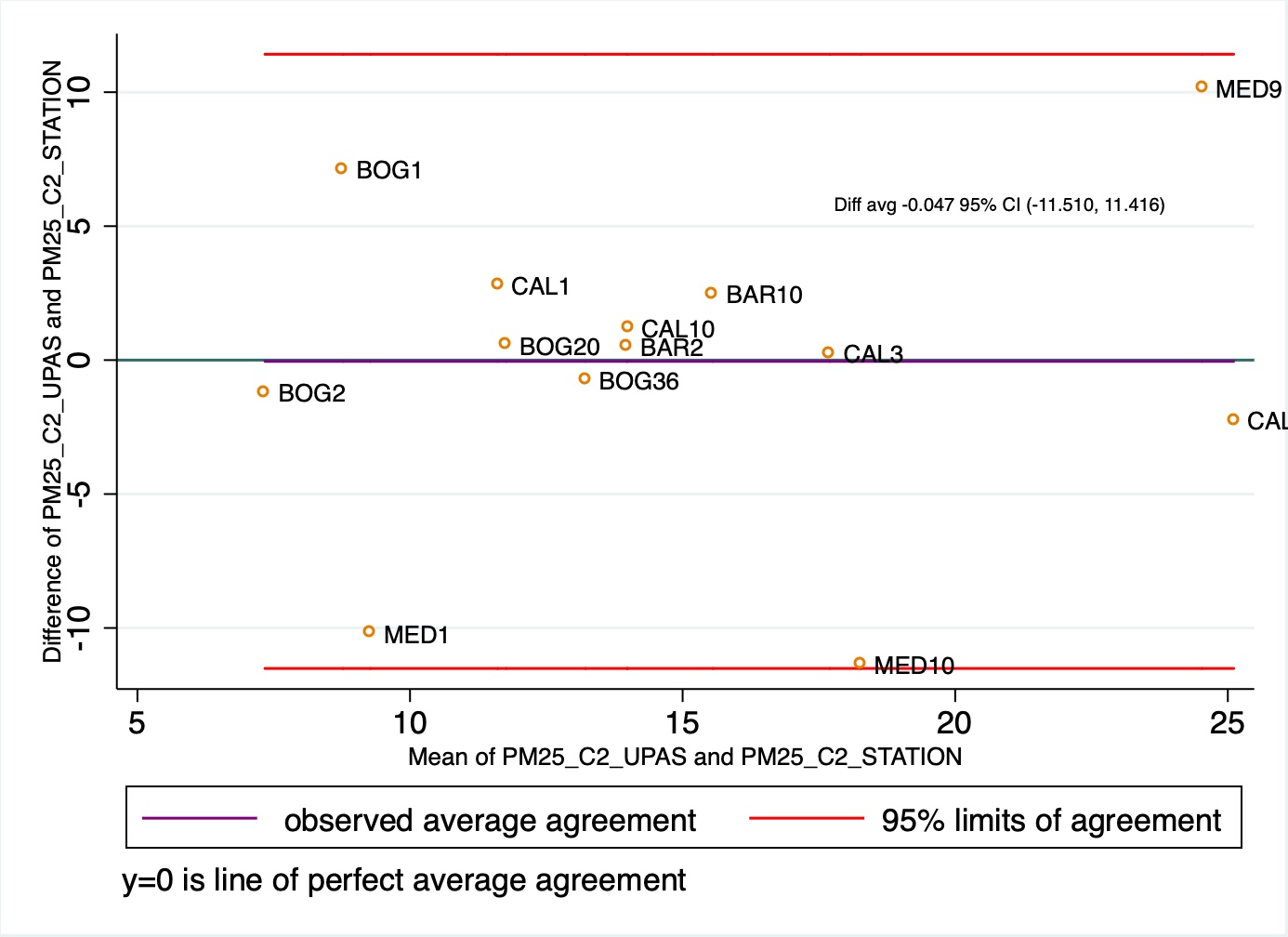 |

Figure S2. Measured versus predicted  PM_2.5_ concentrations (μg/m^3^) for LUR annual models for five cities in Colombia

| Barranquilla  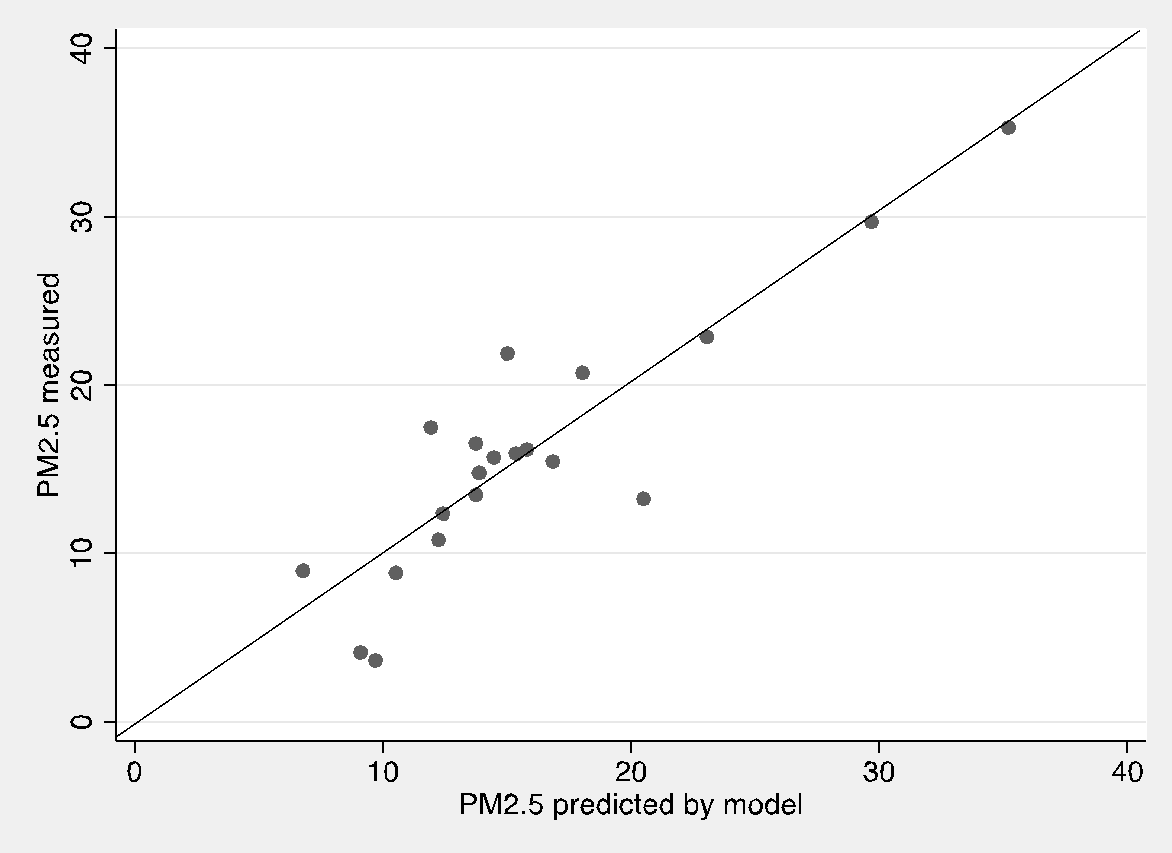 | Bogotá  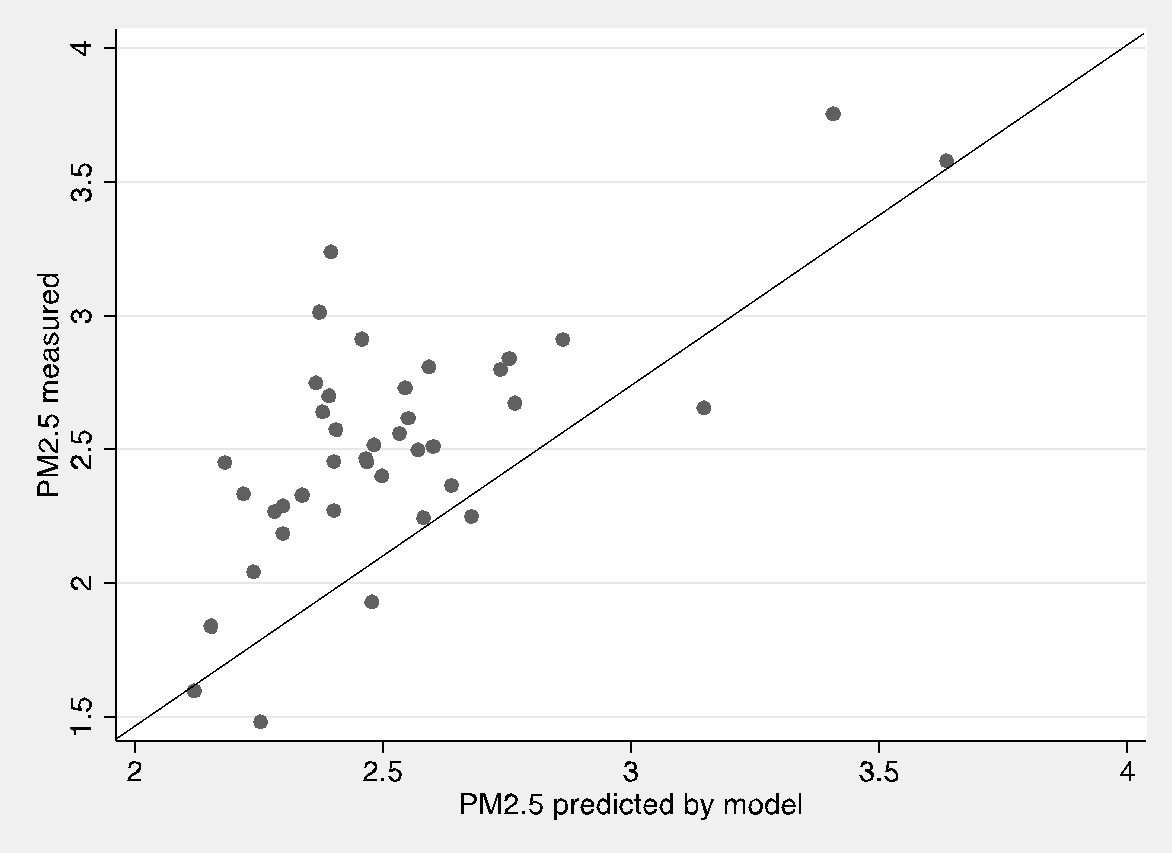 |
| --- | --- |
| Bucaramanga  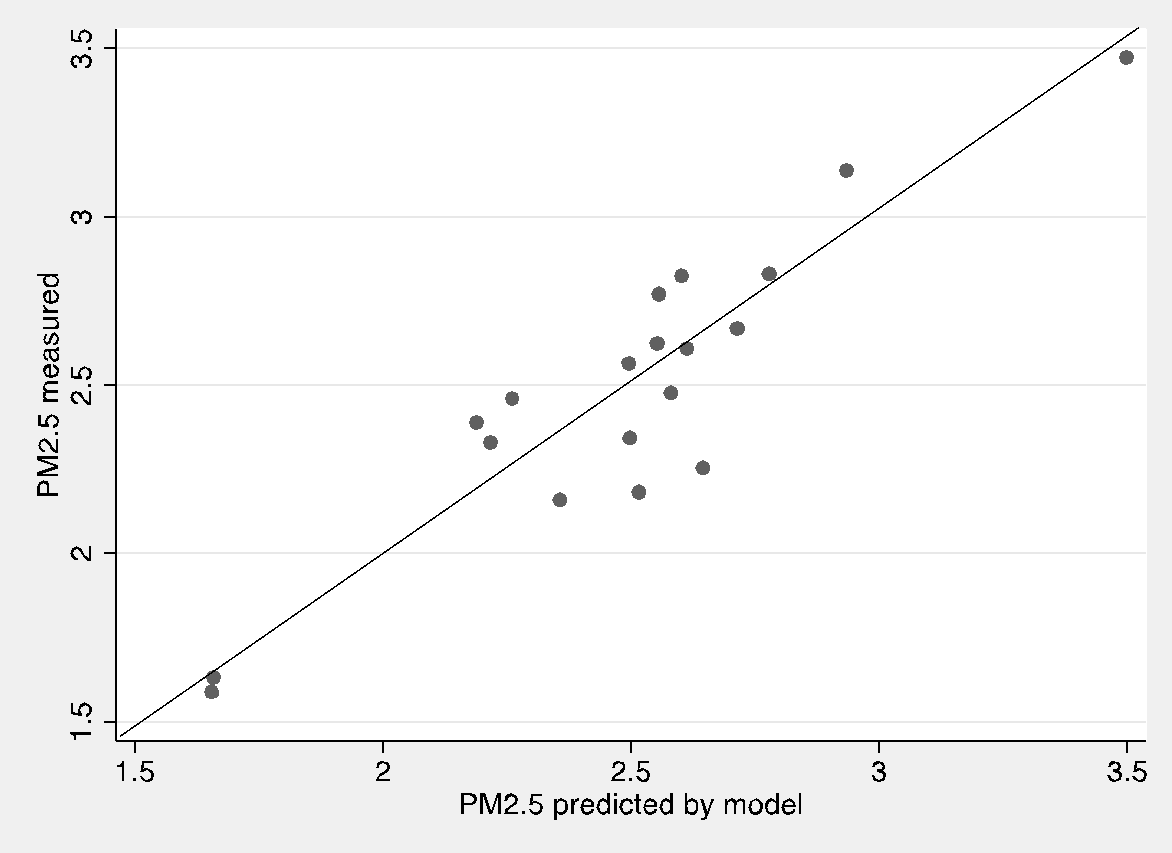 | Cali  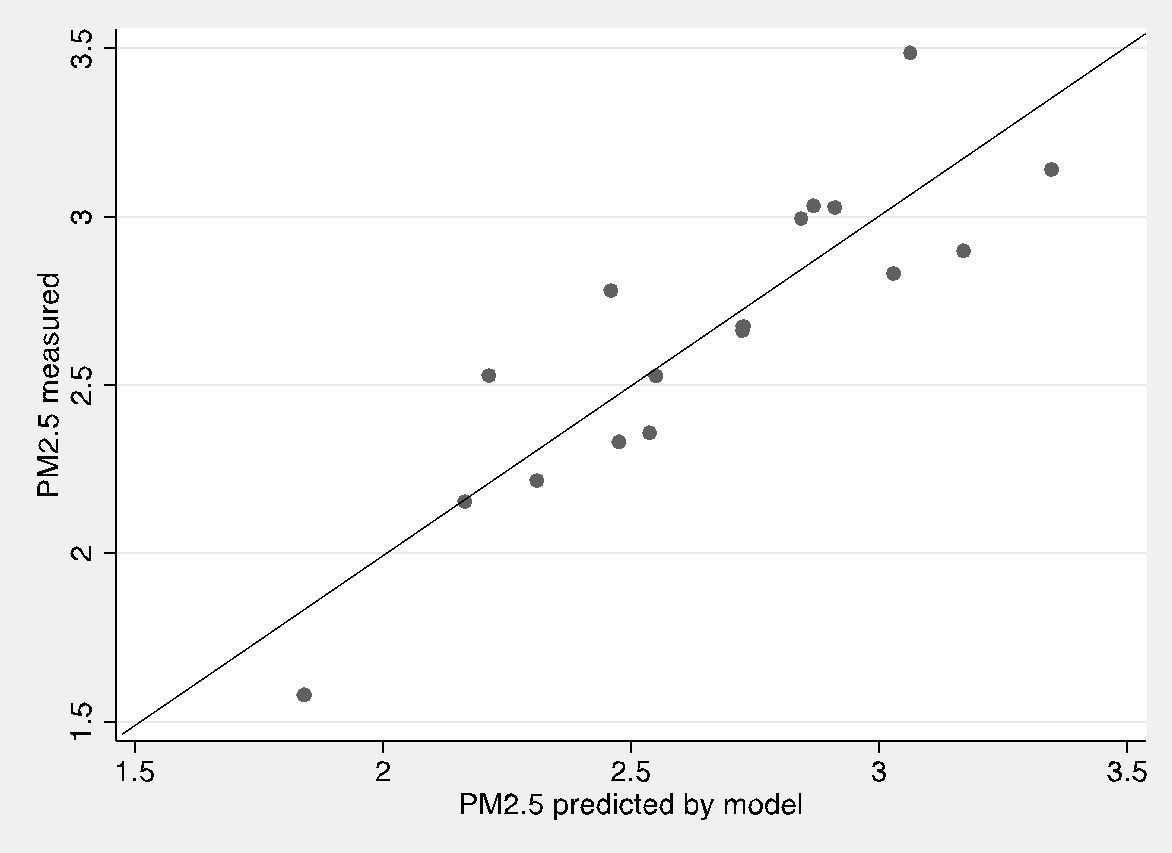 |
| Medellín  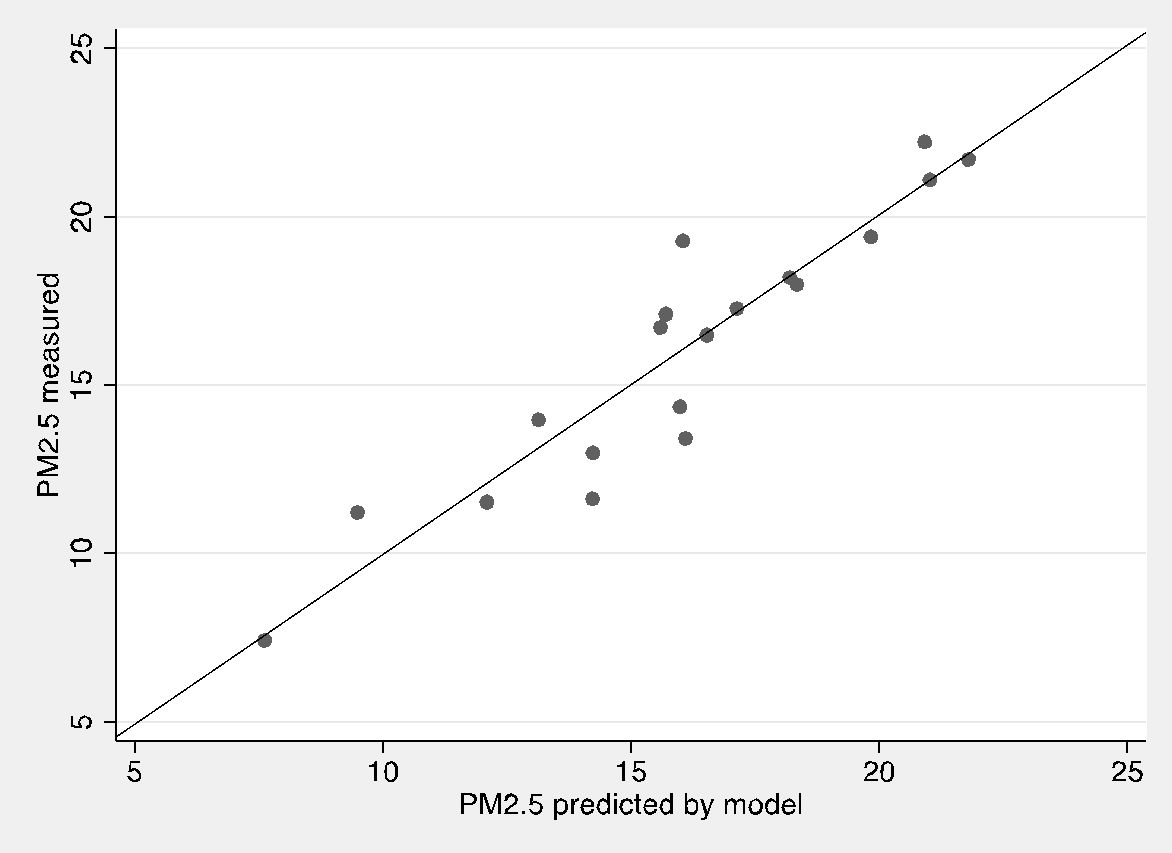 |  |

Figure S3. Measured versus predicted NO_2_ concentrations (μg/m^3^) for LUR annual models for five cities in Colombia

| Barranquilla  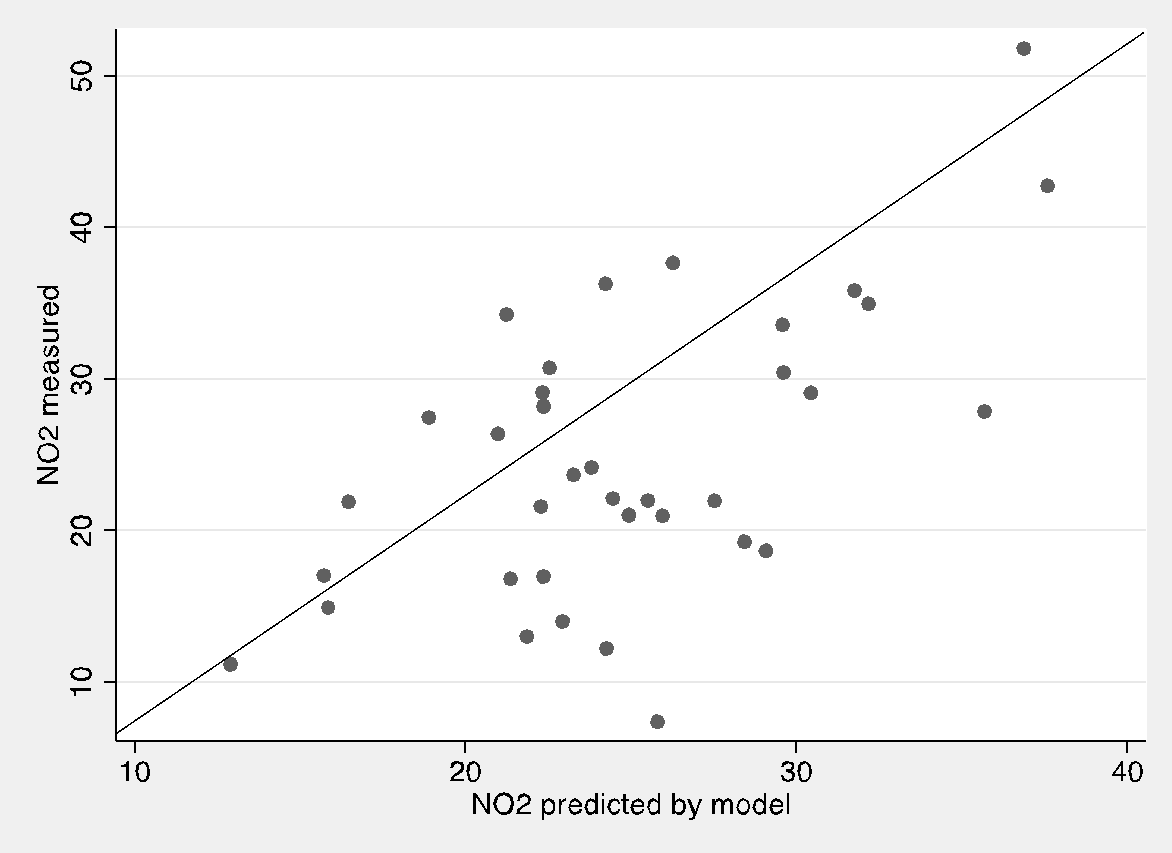 | Bogotá  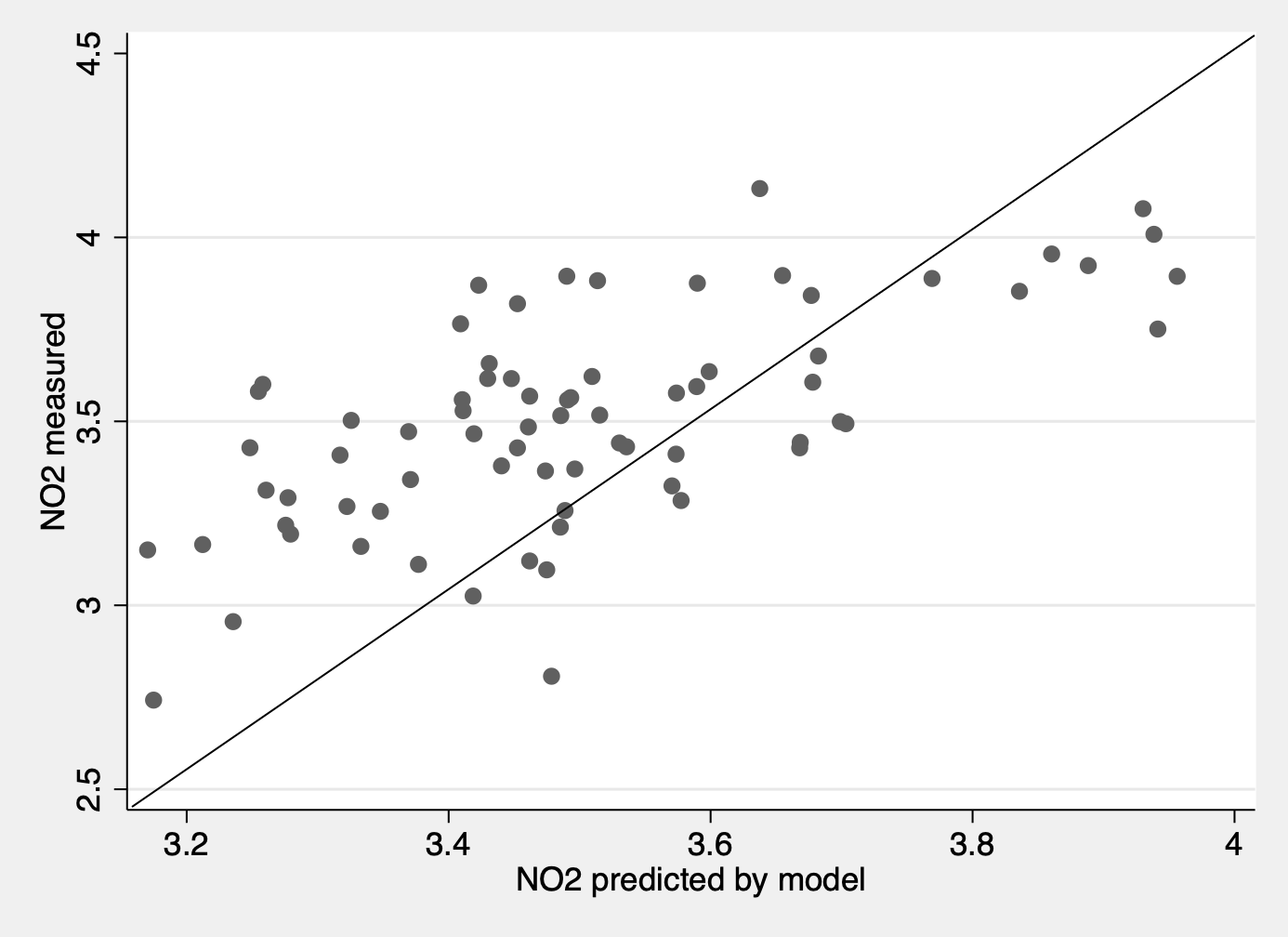 |
| --- | --- |
| Bucaramanga  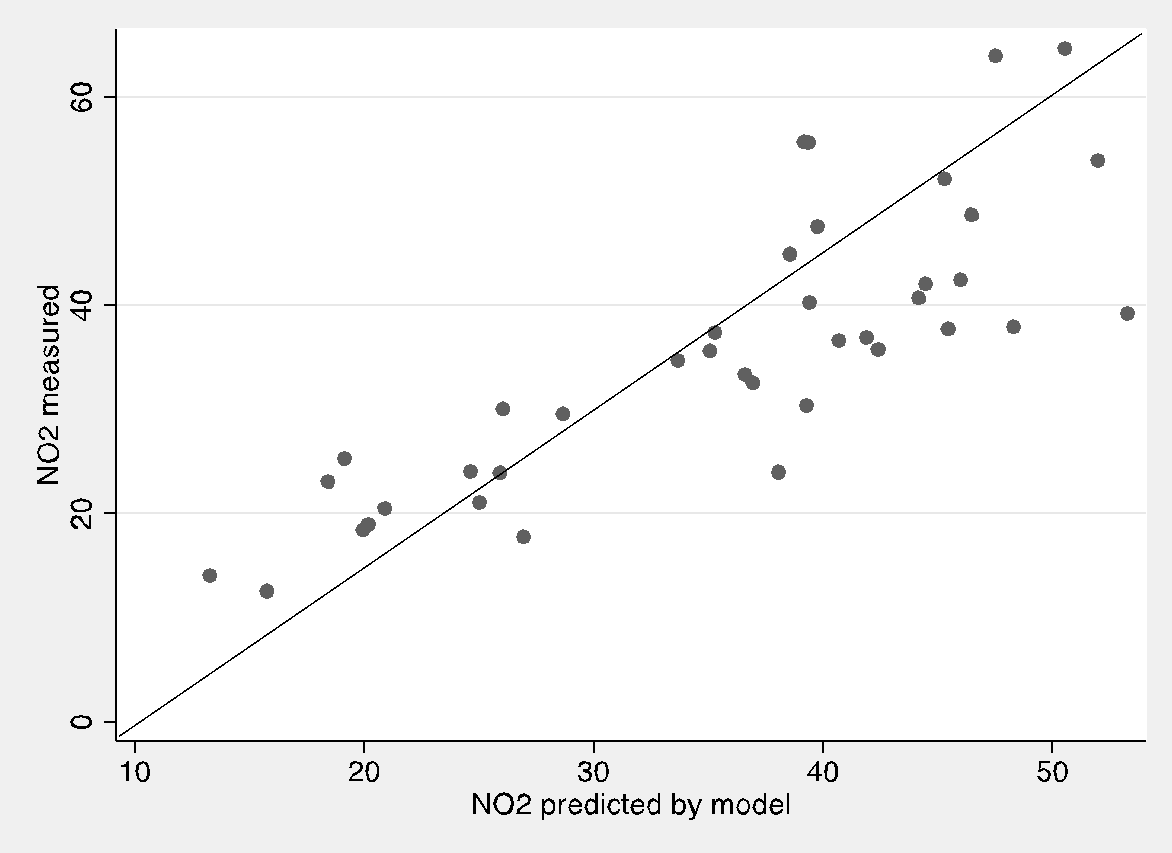 | Cali  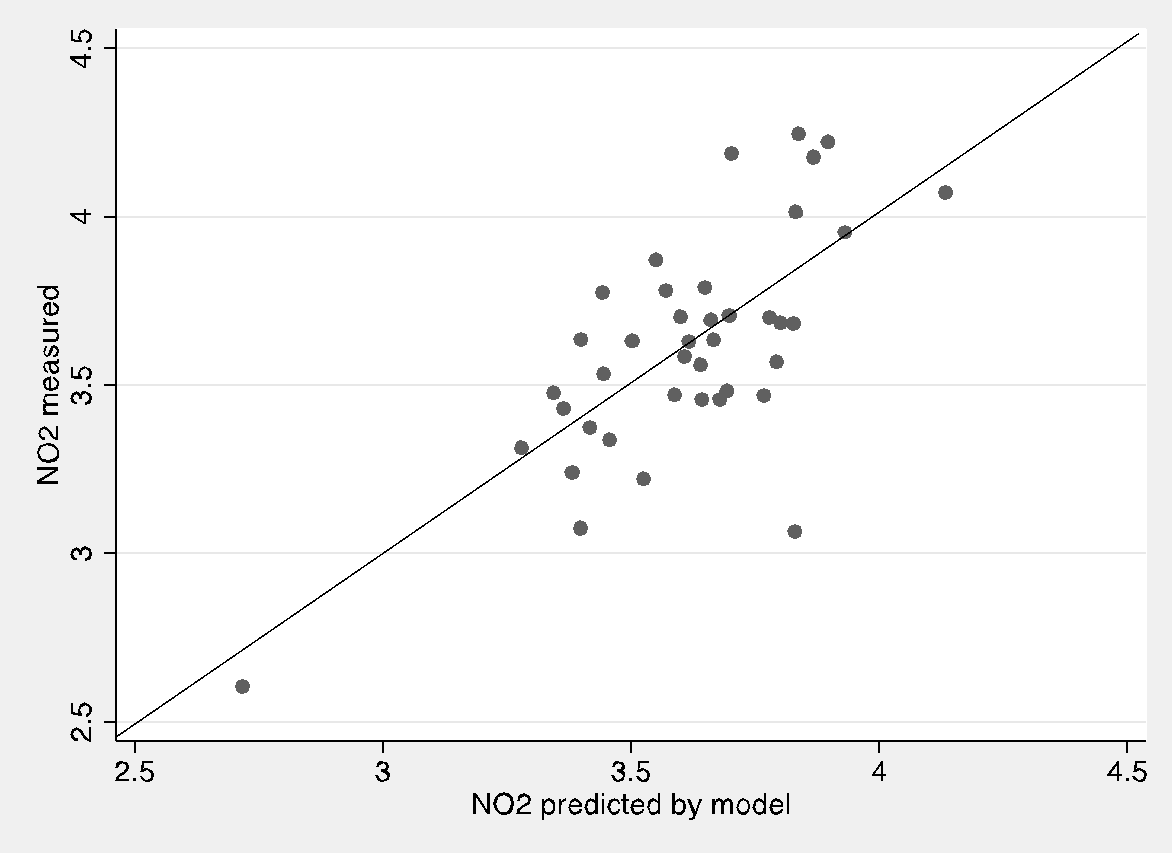 |
| Medellín  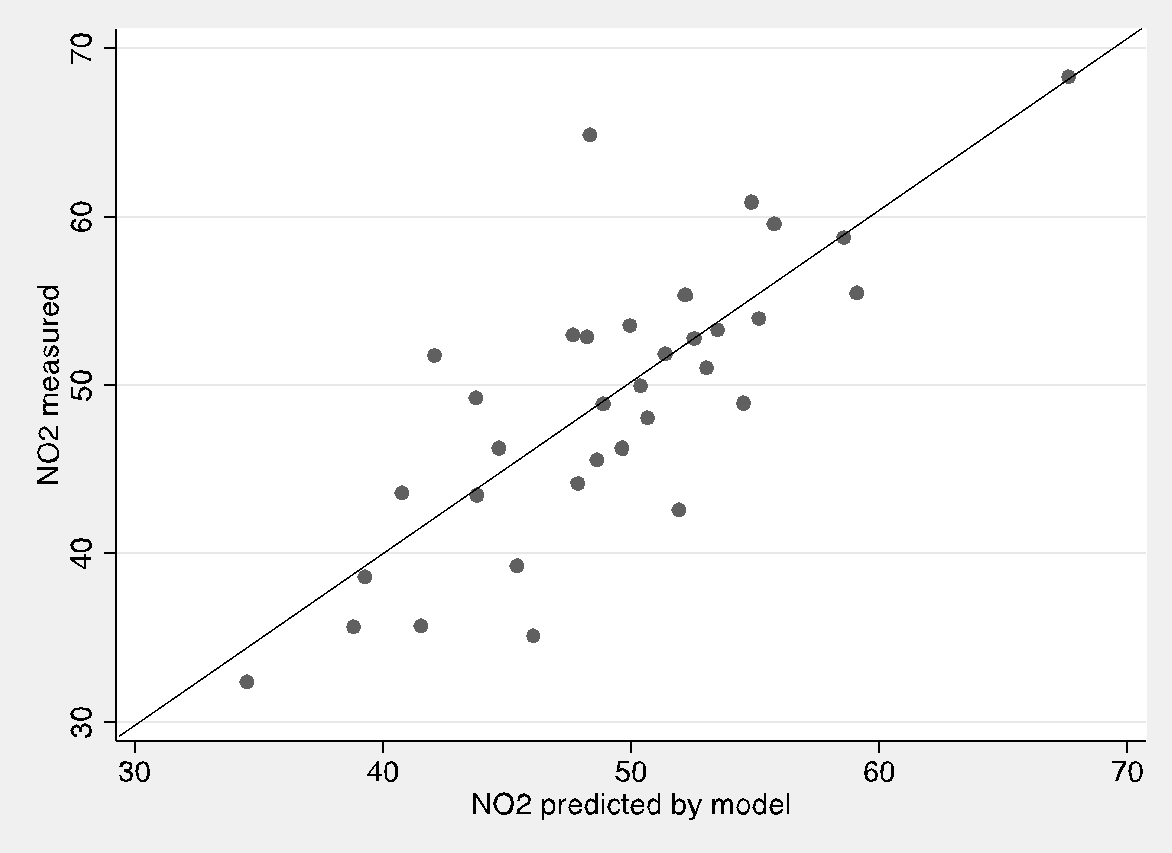 |  |
